# Associations of chrononutrition and sleep with fatigue in multiple sclerosis: an analysis of the Swiss Multiple Sclerosis Registry

**DOI:** 10.1136/bmjno-2025-001471

**Published:** 2026-04-28

**Authors:** Nicholas E Phillips, Vladeta Ajdacic-Gross, Jessica Rebeaud, Guillaume Thévoz, Stefania Iaquinto, Claudia Baum, Christian P Kamm, Jens Kuhle, Irene Rapold, Patrick Roth, Chiara Zecca, Viktor von Wyl, Simone JPM Eussen, Nina Steinemann, Caroline Pot, Tinh-Hai Collet

**Affiliations:** 1 Service of Endocrinology, Diabetology and Metabolism, Department of Medicine, Geneva University Hospitals, Geneva, Switzerland; 2 Laboratories of Neuroimmunology, Center for Research in Neuroscience and Service of Neurology, Department of Clinical Neurosciences, Lausanne University Hospital and University of Lausanne, Lausanne, Switzerland; 3 The Thoracic and Endocrine Surgery Division, Department of Surgery, Geneva University Hospitals, Geneva, Switzerland; 4 Department of Cell Physiology and Metabolism, Faculty of Medicine, University of Geneva, Geneva, Switzerland; 5 Swiss MS Registry, Epidemiology, Biostatistics and Prevention Institute, University of Zurich, Zürich, Switzerland; 6 Department of Epidemiology, Epidemiology, Biostatistics and Prevention Institute, University of Zürich, Zürich, Switzerland; 7 Neurology, Rehabilitation Hospital Zihlschlacht, Zihlschlacht-Sitterdorf, Switzerland; 8 Neurology and Neurorehabilitation Center, Lucerne Cantonal Hospital, Lucerne, Switzerland; 9 Department of Neurology, Bern University Hospital (Inselspital) and University of Bern, Bern, Switzerland; 10 Departments of Biomedicine and Clinical Research, Multiple Sclerosis Centre and Research Center for Clinical Neuroimmunology and Neuroscience (RC2NB), University Hospital Basel and University of Basel, Basel, Switzerland; 11 Department of Neurology, University Hospital Zürich and University of Zürich, Zürich, Switzerland; 12 Department of Neurology, Neurocenter of Southern Switzerland, Lugano Regional Hospital, Lugano, Switzerland; 13 Faculty of Biomedical Sciences, Università della Svizzera italiana, Lugano, Switzerland; 14 Department of Epidemiology; Cardiovascular Research Institute Maastricht (CARIM); and Care and Public Health Research Institute (CAPHRI), Maastricht University Faculty of Health Medicine and Life Sciences, Maastricht, Netherlands; 15 Diabetes Centre, Faculty of Medicine, University of Geneva, Geneva, Switzerland

**Keywords:** MULTIPLE SCLEROSIS, SLEEP

## Abstract

**Background:**

Fatigue is a common symptom of multiple sclerosis (MS) and impacts quality of life, yet the role of chrononutrition and sleep in MS management remains underexplored. We aimed to investigate the cross-sectional associations of meal and sleep timing with fatigue in persons with MS (pwMS).

**Methods:**

We implemented a chrononutrition questionnaire within the Swiss MS Registry to quantify the relationships between meal timing, sleep patterns and fatigue among pwMS.

**Results:**

In unadjusted analyses of 958 participants (median age 49 years, 73% women), a longer interval between the first and last meals (eating window) was associated with less fatigue (OR per 1 SD change in predictor=0.84, 95% CI 0.75 to 0.94), while later wake-up (OR 1.26, 95% CI 1.12 to 1.43) and first meal (OR 1.28, 95% CI 1.14 to 1.44) were associated with more fatigue. Later weekend versus weekday patterns (‘social jetlag’) across meal and sleep timing were associated with less fatigue.

Adjustment for lifestyle factors attenuated most of the relationships, with employment status being the most influential. After adjustment, a larger ‘social jetlag’ in first meal (OR 0.86, 95% CI 0.76 to 0.97) and going to bed later (OR 0.86, 95% CI 0.76 to 0.97) remained associated with less fatigue, while a longer time in bed (OR 1.28, 95% CI 1.12 to 1.46) remained associated with more fatigue.

**Conclusions:**

Associations of meal and sleep timing with fatigue in pwMS were influenced by lifestyle factors, particularly employment status. After adjustment, eating the first meal later during weekends compared with weekdays, going to bed later and a shorter time in bed were associated with less reported fatigue. The apparent benefits of ‘social jetlag’ on first meal times require confirmation from prospective and interventional studies.

## Introduction

 Multiple sclerosis (MS) is a chronic autoimmune condition marked by inflammation and axonal degeneration within the central nervous system.^1^ The prevalence of MS is rising worldwide,^2^ and in Switzerland, the estimated prevalence of 210/100 000 inhabitants in 2021 was 20% higher than in 2016.^3^ MS impacts health-related quality of life, resulting in psychological distress and financial difficulties for affected individuals, relatives and caregivers.^4^ MS-related fatigue has an estimated prevalence of 40%–80% and is among the most common and disabling MS symptoms.^3 5^

In addition to food quality, the timing of food consumption (also termed chrononutrition) has emerged as a potential key factor to control cardiometabolic and neurologic diseases. Meal timing impacts the duration of the fasted state, known to affect circadian rhythms, metabolic and inflammatory processes, leading to cardiovascular conditions.^6^ Intermittent fasting, a dietary regimen involving prolonged periods of fasting, has shown potential in alleviating symptoms in experimental models and preliminary human studies of MS,^7^,^9^ although the role of chrononutrition in MS-related symptoms has to be explored in larger-scale human studies.

‘Social jetlag’ refers to the difference in sleep patterns between weekdays and weekends, reflecting a mismatch between internal circadian rhythms and social cues.^10^ It can be viewed as an indicator of circadian misalignment, disrupting hormonal and metabolic regulation, although possible benefits (eg, weekend sleep debt recovery) may exist.

We aimed to investigate the cross-sectional associations of meal and sleep timing with fatigue using a specifically designed sleep and chrononutrition questionnaire in a survey of people with MS (pwMS). The influence of meal and sleep timing on fatigue could provide valuable insights into non-pharmacological strategies for MS.

## Methods

The Swiss Multiple Sclerosis Registry started in June 2016 and enrols adults cared for MS in Switzerland.^11^

### Clinical data and questionnaires

**Figure 1:**
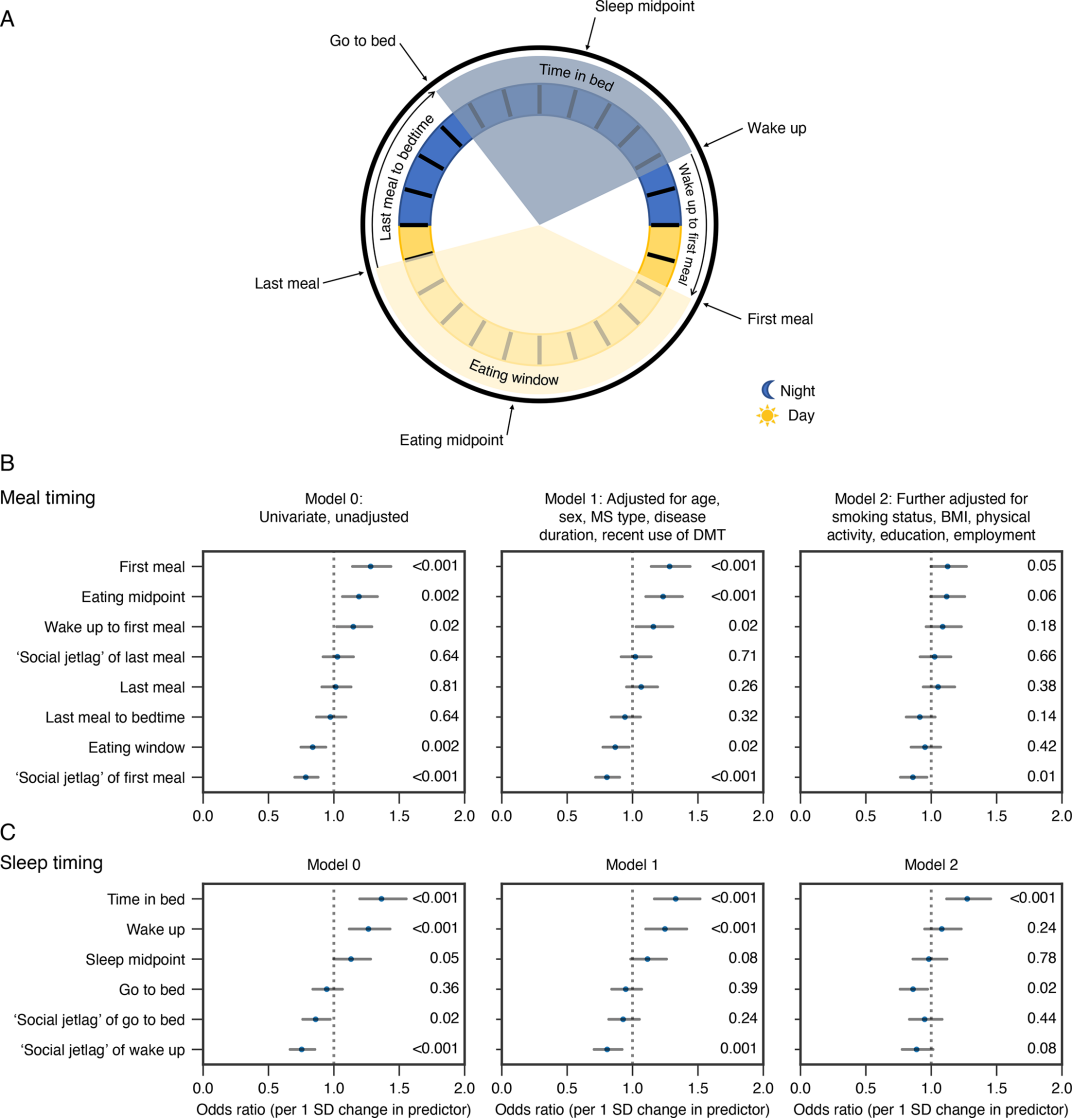
The relationship of meal and sleep timing variables with fatigue. (A) Schematic showing the definition of meal and sleep timing variables (see also online supplemental methods) along the 24-hour clock (black circle), night (blue) and day (yellow). The clock time variables (first meal, last meal, go to bed, wake up times) are shown outside the circle, together with the eating midpoint (midpoint between the first and last meal) and sleep midpoint (midpoint between going to bed and waking up). The period/duration variables are shown inside the circle (eating window, last meal to bed, time in bed, wake up to first meal). (B–C) ORs (dots) and the 95% CIs (lines) from an ordinal logistic regression model showing the association of meal (B) and sleep timing (C) variables with fatigue. ORs reflect the change in odds of being in a higher fatigue quintile per 1 SD increase in the predictor. The ‘social jetlag’ time variables express the weekend minus the weekday value.^10^ P values are shown on the right of each subpanel. BMI, body mass index; DMT, disease-modifying treatment; MS, multiple sclerosis.

At the 36-month follow-up, meal timing was assessed with a questionnaire on eating and sleep rhythms (online supplemental data), while clinical data were from the survey initial questionnaire.^11^ Definitions of variables on eating timing relative to sleep timing are described in figure 1A and online supplemental methods. For time variables of first meal, last meal, going to bed and wake up, we calculated an extra ‘social jetlag’ variable expressing the weekend minus the weekday difference, by analogy with ‘social jetlag’.^10^

Fatigue was assessed using the Modified Fatigue Impact Scale-21, which refers to the past 4 weeks and includes physical (0–36 points), cognitive (0–40 points) and psychosocial dimensions (0–8 points) of fatigue, with higher scores indicating greater burden (total 0–84 points).^12^ Due to its skewed distribution, we categorised the fatigue score into quintiles.

### Statistical analyses

The statistical analysis was restricted to participants with completed fatigue and chrononutrition questionnaires. We normalised each independent variable by its SD and report OR per 1 SD change in predictor. In our first model (model 0), we used univariate ordinal logit regression using all available data, with fatigue as the dependent variable and food or sleep timing variables as the independent variable. We then adjusted for confounding variables, with model 1 adjusting for age, sex, MS type, disease duration and use of disease-modifying treatment within the last 6 months, and model 2 additionally adjusting for smoking status, body mass index, physical activity, education level and current employment status. We used multivariate imputation by chained equations with predictive mean matching to impute missing data (0.0–13.7% fraction), with 25 burn-in iterations and 50 imputations (online supplemental methods). We used a change-in-estimate approach to rank confounders by their influence on the adjusted regression coefficients, where we iteratively removed each of the confounders from the fully adjusted model (model 2) to observe the change in the inferred coefficients.

## Results

Out of the 958 included participants, 696 (72.8%) were women, 623 (65.1%) had relapsing-remitting MS, 201 (21.0%) had secondary progressive MS, and the median age was 49 (IQR 40–58) years (online supplemental table 1).

Definitions of food and sleep timing variables are shown in figure 1A. Strong correlations were observed among several variables, indicating overlapping information between exposures (online supplemental figure 1). Accordingly, exposures were examined in univariable models to minimise instability due to multicollinearity. In an unadjusted analysis, a later first meal (OR 1.28, 95% CI 1.14 to 1.44) and a later eating midpoint (OR 1.19, 95% CI 1.07 to 1.33) were associated with higher levels of fatigue (figure 1B, online supplemental table 2). A longer eating window was associated with lower fatigue levels (OR 0.84, 95% CI 0.75 to 0.94), and individuals who ate their first meal later during the weekend compared with weekdays (ie,‘social jetlag’ of first meal) also reported lower fatigue levels (OR 0.79, 95% CI 0.70 to 0.88). In contrast to the first meal, fatigue levels were not associated with the time of the last meal (OR 1.01, 95% CI 0.91 to 1.13) or its difference between weekdays and weekends (‘social jetlag’ of last meal, OR 1.03, 95% CI 0.92 to 1.15). This pattern was equally observed for the time from waking to the first meal and from the last meal to bedtime, where a longer period between waking and eating was associated with higher fatigue (OR 1.15, 95% CI 1.02 to 1.29), but there was no association with the time between the last meal and going to bed (OR 0.97, 95% CI 0.87 to 1.09).

Among sleep variables, time in bed (OR 1.36, 95% CI 1.20 to 1.55) and wake-up time (OR 1.26, 95% CI 1.12 to 1.43) were associated with higher fatigue levels (figure 1C, online supplemental table 3). ‘Social jetlag’ in go to bed (OR 0.86, 95% CI 0.76 to 0.97) and wake-up times (OR 0.75, 95% CI 0.66 to 0.85) correlated with lower fatigue, indicating that individuals going to bed and waking up later during weekends compared with weekdays had lower average fatigue levels.

When examining whether these unadjusted associations were driven by demographics or MS-related variables, we found no substantial differences compared with the original unadjusted model (model 1 vs model 0, figure 1B,C). The fully adjusted analysis, also accounting for lifestyle factors, generally reduced the magnitude of the inferred coefficients (model 2, figure 1B,C, online supplemental tables 2 and 3). The magnitude of the first meal effect was reduced (OR 1.13, 95% CI 1.00 to 1.27), but its ‘social jetlag’ remained the strongest association after adjustment (OR 0.86, 95% CI 0.76 to 0.97). Among the sleep variables, the time in bed (OR 1.28, 95% CI 1.12 to 1.46) was associated with higher fatigue levels, and go to bed time (OR 0.86, 95% CI 0.76 to 0.97) was associated with lower fatigue levels, even after adjustment.

Given that most of the effect sizes were reduced with full adjustment in model 2, we examined which confounding variable contributed most to this reduction. The change-in-estimate approach revealed that employment status had the largest contribution across all confounders in reducing the magnitude of the effect (online supplemental figure 2). Stratifying by employment status revealed that ‘social jetlag’ of first meals may only be relevant for working individuals (online supplemental figure 3).

## Discussion

Fatigue is a prevalent and debilitating symptom of MS impacting quality of life. Associations of meal and sleep timing with fatigue in pwMS were strongly influenced by lifestyle factors, particularly employment status. After adjustment, eating the first meal later during weekends, going to bed later and a shorter time in bed were all associated with less fatigue. Due to the possible reverse causation in this cross-sectional design, that is, fatigue affecting sleep and meal timing (discussed below), cautious interpretation of these associations is warranted.

Given the potential benefits of time-restricted eating in MS,^6^ it was unexpected that participants who ate early and had a longer eating window had less fatigue in the unadjusted analysis. However, our findings are consistent with our recent case-control study, where pwMS had a shorter eating window than control participants.^13^ Although the clinical context differed, a longer eating window was linked to reduced insomnia and inflammation in colorectal cancer survivors.^14^ This suggests that, while much of chrononutrition research focuses on narrowing the eating window, expanding it may have potential benefits in specific clinical settings. We thus recommend a cautious approach to any future intermittent fasting interventions (eg, time-restricted eating) that delay and narrow the eating window in pwMS.

‘Social jetlag’ is viewed as an indicator of circadian misalignment.^10^ It was therefore unexpected that currently working participants who woke up and ate later during weekends reported less fatigue. This may suggest that varying weekend vs weekday routines offers physical, social and psychological benefits, supporting a more adaptive approach to daily living and promoting rest on weekends.

Despite the large cohort size, the primary limitation of our study is its cross-sectional design, making determination of causality challenging. If significant reverse causation is present, it would imply that fatigue disrupts eating and sleeping timing. This could nonetheless remain a clinically useful finding: if fatigue disrupts eating and sleeping patterns among pwMS, then adaptation of social systems and workplace policies may be required. Second, residual confounding by unmeasured factors influencing fatigue is possible, e.g. undiagnosed sleep or mental health conditions. Self-reported measures of fatigue, sleep and eating are subject to recall and reporting biases. Participants taking part in the survey may differ from those with more severe fatigue or disability, potentially limiting external validity. Thirdly, the fatigue score, the meal and sleep times refer to the past 4 weeks. We cannot conclude on the daily estimates and variability in fatigue, meal and sleep times. Finally, irregular working hours influence fatigue, but this information was not collected in the survey questionnaire of this study.

Given the growing evidence that fasting duration impacts multiple physiological processes, meal timing is emerging as a promising parameter in fatigue of pwMS.^9^ Future studies could consider a crossover meal-timing intervention or a prospective cohort with repeated measurements of meal and sleep timing, fatigue, cognitive function to evaluate temporal ordering and within-person changes. Additionally, intermittent fasting trials among pwMS should exercise caution when delaying the first meal. The apparent benefits of ‘social jetlag’ in wakeup and first meal times require further investigation and may yield novel interventions based on the promotion of social flexibility.

## Supplementary material

10.1136/bmjno-2025-001471 online supplemental file 1
