# Supplementary material for: Associations of chrononutrition and sleep with fatigue in multiple sclerosis: an analysis of the Swiss Multiple Sclerosis Registry
Source: BMJ Neurol Open. 2026 Apr 28;8(1):e001471. doi: 10.1136/bmjno-2025-001471 (PMC13141214; doi:10.1136/bmjno-2025-001471)
Supplement: online supplemental file 1 [file bmjno-8-1-s001.docx]

**Supplementary Methods**

*Questionnaire data*

The Swiss Multiple Sclerosis (MS) Registry surveys were provided in three languages (German, French and Italian) and could be completed using either online or pen-and-paper formats [1]. Participants entered the surveys by completing a short initial questionnaire followed by a comprehensive baseline questionnaire. Further questionnaires followed semiannually and were confined to those diagnosed with MS.

*Other measurements*

Confounders were selected *a priori* based on domain expertise and included variables likely related to both the exposure (sleep and meal timing) and/or the outcome (fatigue). The confounding variables used in the statistical models were either derived from baseline information or obtained from the 36-month follow-up (FU36) questionnaire of the Swiss MS Registry survey [1]. Age was calculated as the difference between the birth year and the date of completion of the questionnaire, and disease duration as the time between the date of MS diagnosis reported and the questionnaire date.

Baseline-derived variables include body mass index (BMI) calculated from self-reported weight (kg) and squared height (m^2^); smoking status assessed as a categorical variable with three response options (never, former, current), with former and current smokers combined into an ‘ever smoker’ binary variable; and highest completed education assessed as a categorical variable with predefined response options (incomplete compulsory school, completed compulsory school, vocational training, upper secondary education [‘Gymnasium’ or ’Lycée’], higher professional education, university, applied university, others), and dichotomized into ‘below college level’ (the first 4 categories) and ‘college level and above’.

FU36-derived variables include MS type assessed as a categorical variable with predefined response options (clinically isolated syndrome [CIS], relapsing-remitting MS [RRMS], secondary progressive MS [SPMS], primary progressive MS [PPMS], transition between disease stages), with CIS and transitions grouped as ‘Other’; use of disease-modifying therapies (DMT) assessed for the preceding six months using predefined response options for each listed DMT (initiation, ongoing use, pause, or discontinuation); physical activity, assessed the number of days (possible range 0-7) in the previous week with ≥ 30 minutes of activity causing slightly increased breathing; and employment status, assessed as a binary variable (‘currently working’ or ‘not working’).

*Eating and sleep timing variables*

From the eating and sleep rhythms questionnaire, participants indicated the timing of sleep (waking up, going to bed) and meals (breakfast, lunch, dinner) for both weekdays and weekends. The eating window was defined as the time elapsed between the first to last meal, while the eating midpoint was defined based on the midpoint between the first and last meals (depicted in **Figure 1A**). We calculated the mean weekly value (using a weighted average of 5/7 for weekday and 2/7 for weekend values). The ‘social jetlag’ was calculated as the weekend minus the weekday value for the time variables of first meal, last meal, go to bed and wake up, extending the ‘social jetlag’ concept, first described in the Munich Chronotype Questionnaire to include both sleep and food timing [2].

*Missing data and multiple imputation*

While the total fraction of missing data was small (0.0 to 13.7%), we imputed missing data using multivariate imputation by chained equations (‘MICE’ function, within the Python package ‘statmodels’ v0.13.0) using five nearest neighbours during predictive mean matching (k_pmm=5), Broyden–Fletcher–Goldfarb–Shanno optimization (method 'bfgs'), a burn-in of 25 iterations, and 50 imputations. Variables were imputed with a linear or logistic regression, depending on whether they were continuous or binary, respectively.

**Supplementary Table 1. Population characteristics**

| **Characteristics** | **Median (IQR), or n (%)** | **Mean ± SD** | **% missing data** |
| --- | --- | --- | --- |
| Demographics |  |  |  |
| Age (years) | 49 (40 – 58) | 49.2 ± 12.5 | 0.3% |
| Female | 696 (72.8%) |  | 0.2% |
| Employment status: Currently working | 588 (61.5%) |  | 0.2% |
| High education | 490 (54.9%) |  | 6.9% |
| MS-related |  |  |  |
| Type |  |  | 0.1% |
| Relapsing-remitting MS | 623 (65.1%) |  |  |
| Secondary progressive MS | 201 (21.0%) |  |  |
| Primary progressive MS | 70 (7.3%) |  |  |
| Other | 63 (6.6%) |  |  |
| Disease duration (years) | 10.0 (4.0 – 18.0) | 11.9 ± 9.2 | 2.1% |
| Recent use of disease-modifying treatment | 690 (72.3%) |  | 0.4% |
| Clinical information |  |  |  |
| Body mass index (kg/m^2^) | 23.9 (21.3 – 27.3) | 24.8 ± 5.0 | 6.4% |
| Smoking status: Ever smoked | 497 (53.9%) |  | 3.8% |
| Physical activity (days per week) | 3.0 (2.0 – 5.0) | 3.3 ± 2.2 | 2.7% |
| Fatigue (MFIS-21 score) | 28 (13 – 43) | 28.8 ± 19.0 | 0.0% |
| Sleep |  |  |  |
| Go to bed (hh:mm) | 22:26 (22:00 – 23:00) | 22:27 ± 00:59 | 9.5% |
| Sleep midpoint (hh:mm) | 02:36 (02:09 – 03:11) | 02:41 ± 00:53 | 13.7% |
| Wake up (hh:mm) | 06:47 (06:13 – 07:30) | 06:56 ± 01:10 | 6.1% |
| Time in bed (h) | 8.4 (7.8 – 9.0) | 8.5 ± 1.1 | 13.7% |
| ‘Social jetlag’ of go to bed (h) | 0.0 (0.0 – 0.5) | 0.4 ± 0.6 | 9.5% |
| ‘Social jetlag’ of wake up (h) | 1.0 (0.5 – 2.0) | 1.3 ± 1.4 | 6.1% |
| Chrononutrition |  |  |  |
| First meal (hh:mm) | 08:09 (07:13 – 09:38) | 08:42 ± 02:04 | 0.0% |
| Eating midpoint (hh:mm) | 13:24 (12:45 – 14:19) | 13:33 ± 01:28 | 0.0% |
| Last meal (hh:mm) | 18:30 (17:51 – 19:21) | 18:24 ± 01:56 | 0.0% |
| Eating window (h) | 10.4 (8.4 – 11.5) | 9.7 ± 2.7 | 0.0% |
| ‘Social jetlag’ of first meal (h) | 1.0 (0.0 – 2.0) | 1.0 ± 2.0 | 0.0% |
| ‘Social jetlag’ of last meal (h) | 0.0 (-0.5 to +0.5) | -0.2 ± 2.2 | 0.0% |
| Wake up to first meal (h) | 1.0 (0.5 – 2.4) | 1.8 ± 1.9 | 7.6% |
| Last meal to bedtime (h) | 3.8 (3.0 – 4.5) | 4.0 ± 1.7 | 9.7% |

Footnotes: Categorical variables are reported as total numbers for all available data (% of population), and continuous variables are reported as median (IQR). The mean ± SD column is provided for easier interpretation of modelling (see **Figure 1** and text). Time measures are expressed as clock time (hh:mm), while period/duration measures are expressed as decimal hours. The ‘social jetlag’ time variables express the weekend minus the weekday value [2]. High education includes university, applied university, higher professional education and non-mandatory upper secondary school (‘Gymnasium’ or ’Lycée’). Abbreviations: IQR, Interquartile Range; MFIS-21, Modified Fatigue Impact Scale (total range 0–84 points); MS, Multiple Sclerosis; SD, Standard Deviation.

**Supplementary Table 1b. Population characteristics, by employment status**

| **Characteristics** | **Currently working (n = 588)** | | **Not working (n = 368)** | |
| --- | --- | --- | --- | --- |
|  | **Median (IQR),**  **or n (%)** | **Mean ± SD** | **Median (IQR),**  **or n (%)** | **Mean ± SD** |
| Demographics |  |  |  |  |
| Age (years) | 45 (36 – 53) | 44.3 ± 10.4 | 58 (49 – 65) | 56.9 ± 11.7 |
| Female | 428 (72.8%) |  | 267 (73.0%) |  |
| High education | 339 (61.4%) |  | 149 (44.1%) |  |
| MS-related |  |  |  |  |
| Type |  |  |  |  |
| Relapsing-remitting MS | 454 (77.2%) |  | 168 (45.8%) |  |
| Secondary progressive MS | 67 (11.39%) |  | 133 (36.2%) |  |
| Primary progressive MS | 31 (5.3%) |  | 39 (10.6%) |  |
| Other | 36 (6.1%) |  | 26 (7.1%) |  |
| Disease duration (years) | 7.0 (3.0 – 13.0) | 9.0 ± 7.2 | 15.0 (9.0 – 22.2) | 16.4 ± 10.2 |
| Recent use of DMT | 456 (77.8%) |  | 232 (63.4%) |  |
| Clinical information |  |  |  |  |
| Body mass index (kg/m^2^) | 23.7 (21.4 – 26.6) | 24.7 ± 4.8 | 24.5 (21.3 – 28.2) | 25.1 ± 5.2 |
| Smoking status: Ever smoked | 280 (49.3%) |  | 217 (61.7%) |  |
| Physical activity (days per week) | 3.0 (2.0 – 5.0) | 3.4 ± 2.1 | 3.0 (1.0 – 5.0) | 3.1 ± 2.3 |
| Fatigue (MFIS-21 score) | 22 (9 – 38) | 24.5 ± 18.4 | 36 (24 – 48) | 35.6 ± 17.8 |
| Sleep |  |  |  |  |
| Go to bed (hh:mm) | 22:17 (22:00 – 22:51) | 22:23 ± 00:54 | 22:30 (22:00 – 23:00) | 22:34 ± 01:06 |
| Sleep midpoint (hh:mm) | 02:28 (02:04 – 02:58) | 02:32 ± 00:48 | 02:51 (02:21 – 03:28) | 02:55 ± 00:57 |
| Wake up (hh:mm) | 06:34 (06:04 – 07:09) | 06:43 ± 01:05 | 07:13 (06:34,08:00) | 07:18 ± 01:11 |
| Time in bed (h) | 8.2 (7.7 – 8.8) | 8.3 ± 1.0 | 8.7 (8.1 – 9.5) | 8.7 ± 1.3 |
| ‘Social jetlag’ of go to bed (h) | 0.5 (0.0 – 1.0) | 0.5 ± 0.6 | 0.0 (0.0 – 0.5) | 0.2 ± 0.5 |
| ‘Social jetlag’ of wake up (h) | 1.5 (1.0 – 2.5) | 1.7 ± 1.5 | 0.5 (0.0 – 1.0) | 0.7 ± 1.0 |
| Chrononutrition |  |  |  |  |
| First meal (hh:mm) | 07:51 (07:04 – 09:13) | 08:27 ± 02:00 | 08:39 (07:39 – 10:00) | 09:05 ± 02:06 |
| Eating midpoint (hh:mm) | 13:17 (12:41 – 14:09) | 13:26 ± 01:25 | 13:39 (12:51 – 14:33) | 13:44 ± 01:32 |
| Last meal (hh:mm) | 18:30 (17:59 – 19:21) | 18:25 ± 01:54 | 18:30 (17:51 – 19:21) | 18:22 ± 02:00 |
| Eating window (h) | 10.6 (8.6 – 11.6) | 10.0 ± 2.7 | 9.9 (8.0 – 11.0) | 9.3 ± 2.7 |
| ‘Social jetlag’ of first meal (h) | 1.5 (0.5 – 2.5) | 1.3 ± 2.2 | 0.5 (0.0 – 1.0) | 0.5 ± 1.7 |
| ‘Social jetlag’ of last meal (h) | 0.0 (-0.5 to +0.5) | -0.2 ± 2.3 | 0.0 (-0.5 to 0.0) | -0.3 ± 2.0 |
| Wake up to first meal (h) | 1.0 (0.5 – 2.3) | 1.7 ± 1.8 | 1.0 (0.5 – 2.4) | 1.8 ± 1.9 |
| Last meal to bedtime (h) | 3.6 (3.0 – 4.4) | 3.9 ± 1.7 | 4.0 (3.0 – 4.9) | 4.1 ± 1.7 |

Footnotes: Population characteristics of **Supplementary Table 1**, stratified by employment status. Categorical variables are reported as total numbers for all available data (% of population), and continuous variables are reported as median (IQR). The mean ± SD column is provided for easier interpretation of modelling (see **Figure 1** and text). Time measures are expressed as clock time (hh:mm), while period/duration measures are expressed as decimal hours. The ‘social jetlag’ time variables express the weekend minus the weekday value [2]. High education includes university, applied university, higher professional education and non-mandatory upper secondary school (‘Gymnasium’ or ’Lycée’). Abbreviations: DMT, Disease-Modifying Treatment; IQR, Interquartile Range; MFIS-21, Modified Fatigue Impact Scale (total range 0–84 points); MS, Multiple Sclerosis; SD, Standard Deviation.

**Supplementary Table 1c. Sleep timing and chrononutrition, during weekdays and weekends**

| **Characteristics** | **Weekdays** | | **Weekends** | |
| --- | --- | --- | --- | --- |
|  | **Median (IQR),**  **or n (%)** | **Mean ± SD** | **Median (IQR),**  **or n (%)** | **Mean ± SD** |
| Sleep |  |  |  |  |
| Go to bed (hh:mm) | 22:30 (22:00 – 23:00) | 22:22 ± 01:00 | 22:30 (22:00 – 23:00) | 22:42 ± 01:05 |
| Sleep midpoint (hh:mm) | 02:30 (01:45 – 03:00) | 02:27 ± 00:58 | 03:15 (02:45 – 03:45) | 03:17 ± 00:59 |
| Wake up (hh:mm) | 06:30 (05:30 – 07:00) | 06:34 ± 01:22 | 08:00 (07:00 – 08:30) | 07:52 ± 01:18 |
| Time in bed (h) | 8.0 (7.5 – 9.0) | 8.2 ± 1.3 | 9.0 (8.5 – 10.0) | 9.1 ± 1.3 |
| Chrononutrition |  |  |  |  |
| First meal (hh:mm) | 08:00 (07:00 – 09:30) | 08:25 ± 02:24 | 09:00 (08:30 – 10:00) | 09:24 ± 01:51 |
| Eating midpoint (hh:mm) | 13:15 (12:30 – 14:15) | 13:26 ± 01:40 | 13:45 (13:00 – 14:41) | 13:48 ± 01:31 |
| Last meal (hh:mm) | 18:30 (18:00 – 19:30) | 18:28 ± 02:10 | 18:30 (17:30 – 19:30) | 18:13 ± 02:14 |
| Eating window (h) | 11.0 (8.5 – 12.0) | 10.1 ± 3.1 | 9.5 (7.5 – 10.5) | 8.8 ± 2.8 |
| Wake up to first meal (h) | 1.0 (0.5 – 2.5) | 1.8 ± 2.2 | 1.0 (0.5 – 2.0) | 1.5 ± 1.5 |
| Last meal to bedtime (h) | 3.5 (3.0 – 4.5) | 3.8 ± 1.9 | 4.0 (3.5 – 5.0) | 4.4 ± 2.1 |

Footnotes: Related to **Supplementary Table 1**. Continuous variables are reported as median (IQR) and mean ± SD column for completeness. Time measures are expressed as clock time (hh:mm), while period/duration measures are expressed as decimal hours. Abbreviations: IQR, Interquartile Range; SD, Standard Deviation.

**Supplementary Table 2.** Ordinal regression coefficients for chrononutrition (meal timing) variables

|  | **Model 0: Univariate, unadjusted** | | **Model 1: Adjusted for age, sex, MS type, disease duration, recent use of DMT** | | **Model 2: Further adjusted for smoking status, BMI, physical activity, education, employment** | |
| --- | --- | --- | --- | --- | --- | --- |
| **Predictor** | **β coefficient (95% CI)** | **p-value** | **β coefficient (95% CI)** | **p-value** | **β coefficient (95% CI)** | **p-value** |
| First meal | 0.15 (0.08; 0.22) | <0.001 | 0.15 (0.08; 0.22) | <0.001 | 0.07 (-0.00; 0.14) | 0.05 |
| Eating midpoint | 0.11 (0.04; 0.18) | 0.001 | 0.13 (0.06; 0.20) | <0.001 | 0.07 (-0.00; 0.14) | 0.07 |
| Last meal | 0.01 (-0.06; 0.08) | 0.76 | 0.04 (-0.03; 0.11) | 0.27 | 0.03 (-0.04; 0.09) | 0.47 |
| Eating window | -0.11 (-0.17; -0.04) | 0.002 | -0.09 (-0.16; -0.02) | 0.01 | -0.03 (-0.10; 0.04) | 0.35 |
| ‘Social jetlag’ of first meal | -0.15 (-0.22; -0.08) | <0.001 | -0.13 (-0.20; -0.06) | <0.001 | -0.09 (-0.16; -0.02) | 0.009 |
| ‘Social jetlag’ of last meal | 0.01 (-0.06; 0.08) | 0.76 | 0.01 (-0.06; 0.08) | 0.81 | 0.01 (-0.05; 0.08) | 0.69 |
| Wake up to first meal | 0.09 (0.02; 0.16) | 0.017 | 0.09 (0.02; 0.16) | 0.014 | 0.05 (-0.02; 0.13) | 0.15 |
| Last meal to bedtime | -0.01 (-0.08; 0.06) | 0.71 | -0.03 (-0.11; 0.04) | 0.38 | -0.05 (-0.12; 0.03) | 0.21 |

Footnotes: The ‘social jetlag’ time variables express the weekend minus the weekday value [2]. Abbreviations: BMI, Body Mass Index; CI, Confidence Interval; DMT, Disease-Modifying Treatment; MS, Multiple Sclerosis.

**Supplementary Table 3.** Ordinal regression coefficients for sleep variables

|  | **Model 0: Univariate, unadjusted** | | **Model 1: Adjusted for age, sex, MS type, disease duration, recent use of DMT** | | **Model 2: Further adjusted for smoking status, BMI, physical activity, education, employment** | |
| --- | --- | --- | --- | --- | --- | --- |
| **Predictor** | **β coefficient (95% CI)** | **p-value** | **β coefficient (95% CI)** | **p-value** | **β coefficient (95% CI)** | **p-value** |
| Go to bed | -0.03 (-0.10; 0.04) | 0.41 | -0.03 (-0.10; 0.04) | 0.4 | -0.09 (-0.16; -0.02) | 0.016 |
| Sleep midpoint | 0.08 (0.00; 0.15) | 0.038 | 0.07 (-0.00; 0.14) | 0.067 | -0.01 (-0.09; 0.06) | 0.75 |
| Wake up | 0.13 (0.06; 0.20) | <0.001 | 0.13 (0.06; 0.20) | <0.001 | 0.04 (-0.03; 0.11) | 0.29 |
| Time in bed | 0.17 (0.10; 0.25) | <0.001 | 0.16 (0.09; 0.24) | <0.001 | 0.13 (0.06; 0.21) | <0.001 |
| ‘Social jetlag’ of go to bed | -0.08 (-0.15; -0.01) | 0.031 | -0.04 (-0.11; 0.04) | 0.32 | -0.03 (-0.11; 0.04) | 0.39 |
| ‘Social jetlag’ of wake up | -0.16 (-0.23; -0.09) | <0.001 | -0.12 (-0.20; -0.05) | <0.001 | -0.07 (-0.15; 0.00) | 0.061 |

Footnotes: The ‘social jetlag’ time variables express the weekend minus the weekday value [2]. Abbreviations: BMI, Body Mass Index; CI, Confidence Interval; DMT, Disease-Modifying Treatment; MS, Multiple Sclerosis.

**References to the Supplementary Material**

1 Steinemann N, Kuhle J, Calabrese P, *et al.* The Swiss Multiple Sclerosis Registry (SMSR): study protocol of a participatory, nationwide registry to promote epidemiological and patient-centered MS research. *BMC Neurol*. 2018;18:111. doi: 10.1186/s12883-018-1118-0

2 Roenneberg T, Pilz LK, Zerbini G, *et al.* Chronotype and Social Jetlag: A (Self-) Critical Review. *Biology*. 2019;8:54. doi: 10.3390/biology8030054

**Supplementary Figure 1.**


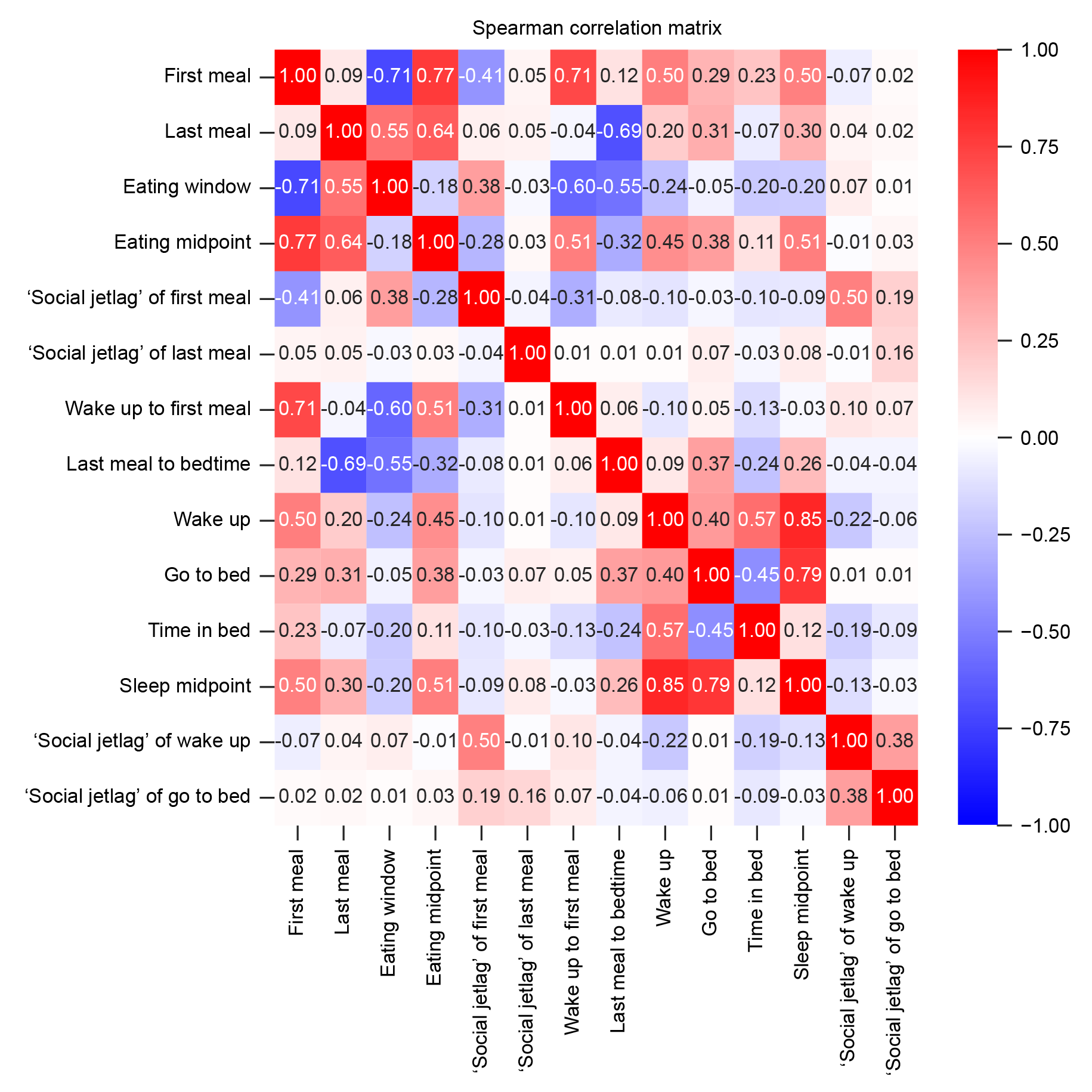


Legend: Spearman rank correlation matrix of meal and sleep timing variables, displayed as a heatmap. Correlation coefficients range from −1.0 (blue) to +1.0 (red), with colour intensity reflecting the strength and direction of the association.

**Supplementary Figure 2.**


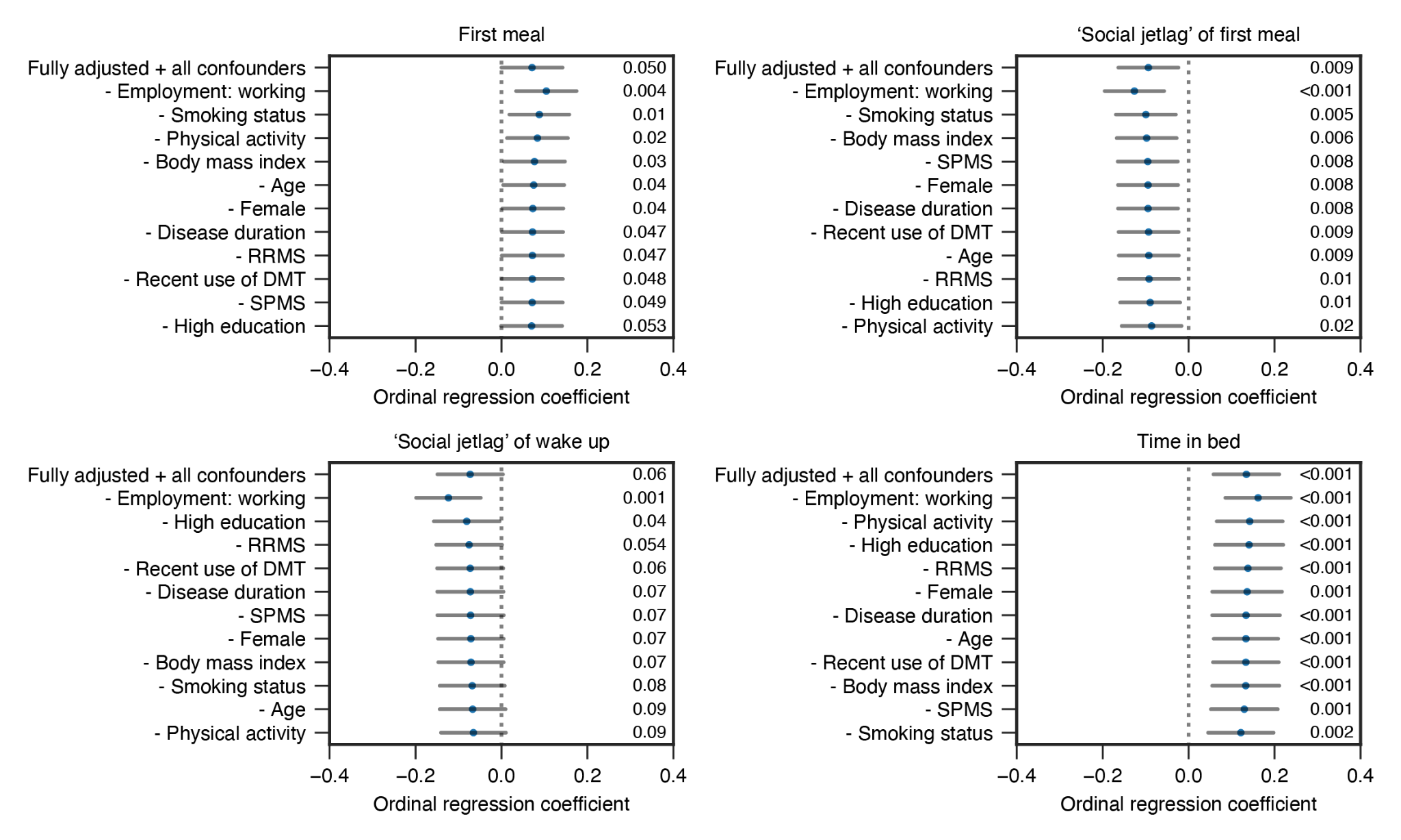


Legend: Change-in-estimate approach to estimate the contribution of each confounder in the fully adjusted model (Model 2). Each confounder variable is iteratively removed from the fully adjusted model, and the regression coefficient is recalculated. The confounders are ranked such that the highest variable shows the strongest effect, i.e. the largest difference compared to the fully adjusted model. The ‘social jetlag’ time variables express the weekend minus the weekday value [2]. Abbreviations: DMT, Disease-Modifying Treatment; RRMS, Relapsing-Remitting Multiple Sclerosis; SPMS, Secondary Progressive Multiple Sclerosis.

**Supplementary Figure 3.**

**
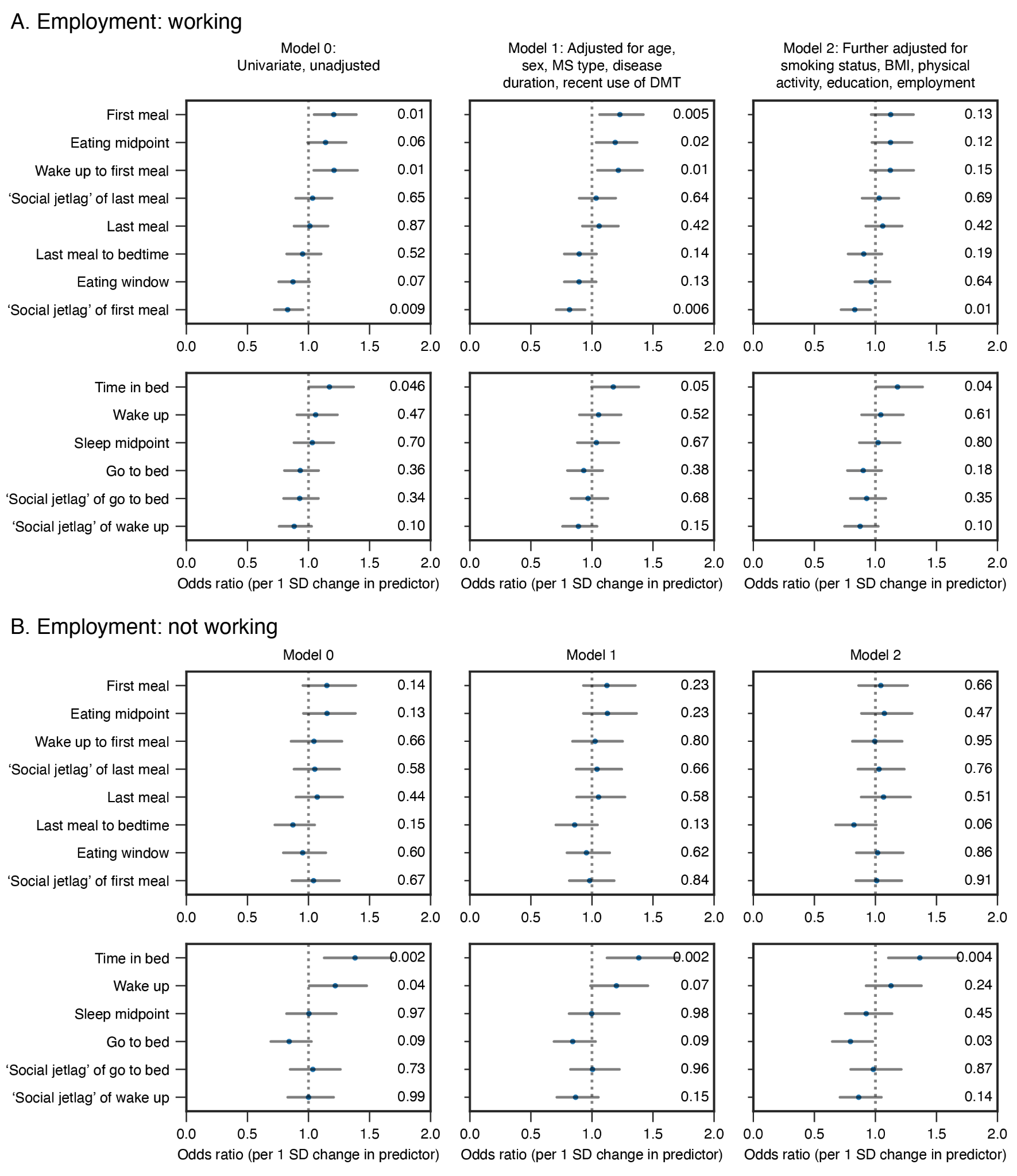
**

Legend: Related to **Figure 1B-C**, stratified analysis by employment status. (A-B) Odds ratios (dots) and the 95% confidence intervals (lines) from an ordinal logistic regression model showing the association of eating and sleep timing variables with fatigue, within the strata of working (A) and non-working (B) participants. Odds ratios reflect the change in odds of being in a higher fatigue quintile per 1 SD increase in the predictor. P-values are shown on the right of each subpanel. Abbreviations: BMI, Body Mass Index; DMT, Disease-Modifying Treatment; MS, Multiple Sclerosis; SD, Standard Deviation.

**Supplementary Data. Food rhythm questionnaire**

This questionnaire aims to assess your usual pattern of food and drink consumption over weekdays and weekends. Part A contains general questions about your eating habits, while Part B includes a table to record your eating and sleeping patterns. Please indicate **all the times you eat and drink**, including morning, afternoon, evening and night snacks. Please note as accurately as possible the averages for weekdays (Monday to Friday) and weekends (Saturday and Sunday). If necessary, you can record several points on the same line (see the penultimate line of the example below). If you tend to eat and drink irregularly, please indicate the average time you eat and drink.

The table below shows an example of how to complete it. If you want to correct your answer, you can draw a large cross over the point you marked in error and then tick the correct point. Completing the questionnaire with the tables will take around 10 minutes.

**EXAMPLE: hours of sleep and food intake in the morning and afternoon during the week
(Monday to Friday)**

|  | **06.00 -06.30** | **06.30 -07.00** | **07.00 -07.30** | **07.30 -08.00** | **08.00 -08.30** | **08.30 -09.00** | **09.00 -09.30** | **09.30 -10.00** | **10.00 -10.30** | **10:30 -11:00** | **11.00 -11.30** | **11.30 -12.00** | **12.00 -12.30** | **12.30 -13.00** | **13.00 -13.30** | **13.30 -14.00** | **14.00 -14.30** | **14.30 -15.00** | **15.00 -15.30** | **15.30 -16.00** | **16.00 -16.30** | **16.30 -17.00** | **17.00 -17.30** | **17.30 -18.00** |
| --- | --- | --- | --- | --- | --- | --- | --- | --- | --- | --- | --- | --- | --- | --- | --- | --- | --- | --- | --- | --- | --- | --- | --- | --- |
|  |  |  |  |  |  |  |  |  |  |  |  |  |  |  |  |  |  |  |  |  |  |  |  |  |
|  |  |  |  |  |  |  |  |  |  |  |  |  |  |  |  |  |  |  |  |  |  |  |  |  |
|  |  |  |  |  |  |  |  |  |  |  |  |  |  |  |  |  |  |  |  |  |  |  |  |  |
| **Wake up time** | 🔾 | 🔾 | ● | 🔾 | 🔾 | 🔾 | 🔾 | 🔾 | 🔾 | 🔾 | 🔾 | 🔾 | 🔾 | 🔾 | 🔾 | 🔾 | 🔾 | 🔾 | 🔾 | 🔾 | 🔾 | 🔾 | 🔾 | 🔾 |
| **Breakfast** | | | | | | | | | | | | | | | | | | | | | | | | |
| Drinks | 🔾 | 🔾 | 🔾 | ● | 🔾 | 🔾 | 🔾 | 🔾 | 🔾 | 🔾 | 🔾 | 🔾 | 🔾 | 🔾 | 🔾 | 🔾 | 🔾 | 🔾 | 🔾 | 🔾 | 🔾 | 🔾 | 🔾 | 🔾 |
| Food | 🔾 | 🔾 | 🔾 | ● | 🔾 | 🔾 | 🔾 | 🔾 | 🔾 | 🔾 | 🔾 | 🔾 | 🔾 | 🔾 | 🔾 | 🔾 | 🔾 | 🔾 | 🔾 | 🔾 | 🔾 | 🔾 | 🔾 | 🔾 |
| **Lunch** | | | | | | | | | | | | | | | | | | | | | | | | |
| Drinks | 🔾 | 🔾 | 🔾 | 🔾 | 🔾 | 🔾 | 🔾 | 🔾 | 🔾 | 🔾 | 🔾 | 🔾 | 🔾 | ● | 🔾 | 🔾 | 🔾 | 🔾 | 🔾 | 🔾 | 🔾 | 🔾 | 🔾 | 🔾 |
| Food | 🔾 | 🔾 | 🔾 | 🔾 | 🔾 | 🔾 | 🔾 | 🔾 | 🔾 | 🔾 | 🔾 | 🔾 | 🔾 | ● | 🔾 | 🔾 | 🔾 | 🔾 | 🔾 | 🔾 | 🔾 | 🔾 | 🔾 | 🔾 |
| **Evening meal** | | | | | | | | | | | | | | | | | | | | | | | | |
| Drinks | 🔾 | 🔾 | 🔾 | 🔾 | 🔾 | 🔾 | 🔾 | 🔾 | 🔾 | 🔾 | 🔾 | 🔾 | 🔾 | 🔾 | 🔾 | 🔾 | 🔾 | 🔾 | 🔾 | 🔾 | 🔾 | 🔾 | 🔾 | ● |
| Food | 🔾 | 🔾 | 🔾 | 🔾 | 🔾 | 🔾 | 🔾 | 🔾 | 🔾 | 🔾 | 🔾 | 🔾 | 🔾 | 🔾 | 🔾 | 🔾 | 🔾 | 🔾 | 🔾 | 🔾 | 🔾 | 🔾 | 🔾 | ● |
| **Morning and afternoon snacks** | | | | | | | | | | | | | | | | | | | | | | | | |
| Water | 🔾 | 🔾 | 🔾 | 🔾 | 🔾 | 🔾 | 🔾 | 🔾 | 🔾 | ● | 🔾 | 🔾 | 🔾 | 🔾 | 🔾 | 🔾 | 🔾 | 🔾 | 🔾 | 🔾 | 🔾 | 🔾 | 🔾 | 🔾 |
| Drinks | 🔾 | 🔾 | 🔾 | 🔾 | 🔾 | 🔾 | 🔾 | ● | 🔾 | 🔾 | ● | 🔾 | 🔾 | 🔾 | 🔾 | 🔾 | 🔾 | 🔾 | 🔾 | 🔾 | 🔾 | 🔾 | 🔾 | 🔾 |
| Food | 🔾 | 🔾 | 🔾 | 🔾 | 🔾 | 🔾 | 🔾 | 🔾 | 🔾 | 🔾 | 🔾 | 🔾 | 🔾 | 🔾 | 🔾 | 🔾 | 🔾 | 🔾 | 🔾 | ● | ● | 🔾 | 🔾 | 🔾 |
|  | | | | | | | | | | | | | | | | | | | | | | | | |
| **Daytime naps** | 🔾 | 🔾 | 🔾 | 🔾 | 🔾 | 🔾 | 🔾 | 🔾 | 🔾 | 🔾 | 🔾 | 🔾 | 🔾 | 🔾 | 🔾 | 🔾 | 🔾 | 🔾 | 🔾 | 🔾 | 🔾 | 🔾 | 🔾 | 🔾 |
|  | **06.00 -06.30** | **06.30 -07.00** | **07.00 -07.30** | **07.30 -08.00** | **08.00 -08.30** | **08.30 -09.00** | **09.00 -09.30** | **09.30 -10.00** | **10.00 -10.30** | **10.30 -11.00** | **11.00 -11.30** | **11.30 -12.00** | **12.00 -12.30** | **12.30 -13.00** | **13.00 -13.30** | **13.30 -14.00** | **14.00 -14.30** | **14.30 -15.00** | **15.00 -15.30** | **15.30 -16.00** | **16:00 -16.30** | **16.30 -17.00** | **17.00 -17.30** | **17.30 -18.00** |

**Part A. Please answer the following questions:**

| 1 | Has your eating pattern (meal and snack times) changed over the last year? |
| --- | --- |

No🔾 Yes🔾

If so, please explain the changes?

................................................................................................................................................................................................. .................................................................................................................................................................................................

| 2 | How many days do you eat **breakfast** during the week?  *(By breakfast, we mean food, not just a cup of coffee or tea).* Please tick the correct answer: |
| --- | --- |

1. During the week (Monday - Friday) 🔾 0 🔾 1 🔾 2 🔾 3 🔾 4 🔾 5

1. If you eat breakfast, do you usually eat it at the same time?

Tick "yes" if you eat breakfast 4 or 5 times a week at the same time:

🔾 no

🔾 yes

1. If you answered "**no**" to question 2b, please explain why:

- working irregular hours
- non-professional activities (sport or leisure) for myself or other members of the family
- other: ............................................................

| 3 | How many days do you eat **breakfast at** the weekend?  *(By breakfast, we mean food, not just a cup of coffee or tea).* Please tick the correct answer: |
| --- | --- |

1. Over the weekend (Saturday and Sunday) 🔾 0 🔾 1 🔾 2

1. If you eat breakfast, do you usually eat it at the same time on Saturday and Sunday?

🔾 no

🔾 yes

1. If you answered "**no**" to question 3b, please explain why:

- working irregular hours
- non-professional activities (sport or leisure) for myself or other members of the family
- other: ..............................................................

| 4 | How many days do you eat **lunch** during the week?  Please tick the correct answer: |
| --- | --- |

1. During the week (Monday - Friday) 🔾 0 🔾 1 🔾 2 🔾 3 🔾 4 🔾 5

1. If you have a midday meal, do you usually eat it at the same time?

Tick "yes" if you eat lunch 4 or 5 times a week at the same time:

🔾 no

🔾 yes

1. If you answered "**no**" to question 4b, please explain why:

- working irregular hours
- non-professional activities (sport or leisure) for myself or other members of the family
- other: ..............................................................

| 5 | How many days do you eat **lunch at** the weekend?  Please tick the correct answer: |
| --- | --- |

1. Over the weekend (Saturday and Sunday) 🔾 0 🔾 1 🔾 2

1. If you eat lunch, do you usually eat it at the same time on Saturday and Sunday?

🔾 no

🔾 yes

1. If you answered "**no**" to question 5b, please explain why:

- working irregular hours
- non-professional activities (sport or leisure) for myself or other members of the family
- other: ............................................................

| 6 | How many days do you eat an **evening meal** during the week?  Please tick the correct answer: |
| --- | --- |

1. During the week (Monday - Friday) 🔾 0 🔾 1 🔾 2 🔾 3 🔾 4 🔾 5
2. If you have an evening meal, do you usually eat it at the same time?
   Tick "yes" if you eat your evening meal 4 or 5 times a week at the same time.

- no
- yes

1. If you answered "**no**" to question 6b, please explain why:

- working irregular hours
- non-professional activities (sport or leisure) for myself or other members of the family
- other: ............................................................

| 7 | How many days do you eat **an evening meal at** the weekend?  Please tick the correct answer: |
| --- | --- |

1. Over the weekend (Saturday and Sunday) 🔾 0 🔾 1 🔾 2
2. If you are having supper: do you usually eat at the same time on Saturday and Sunday?

🔾 no

🔾 yes

1. If you answered "**no**" to question 7b, please explain why:

- working irregular hours
- non-professional activities (sport or leisure) for myself or other members of the family
- other: ............................................................

| 8 | How many days a week do you eat **snacks** (e.g. fruit, biscuits, chocolate)? Please tick the right answer: |
| --- | --- |

1. Over the course of the morning, on 🔾 0 🔾 1 🔾 2 🔾 3 🔾 4 🔾 5 day(s)
2. During the afternoon, on 🔾 0 🔾 1 🔾 2 🔾 3 🔾 4 🔾 5 day(s)
3. Over the course of the evening, on 🔾 0 🔾 1 🔾 2 🔾 3 🔾 4 🔾 5 day(s)

| 9 | How many **snacks** do you eat during the week?  Do you eat these snacks at regular or variable times? |
| --- | --- |

1. During the morning, number of snacks : 🔾 no snack

🔾 1 snack

🔾 more than 1 snack

1. Snack times in the morning: 🔾 always at the same time

🔾 at the same time for 4 out of 5 days

🔾 at varying times



1. During the afternoon, number of snacks : 🔾 no snacks

🔾 1 snack

🔾 more than 1 snack

1. Afternoon snack times : 🔾 always at the same time

🔾 at the same time for 4 out of 5 days

🔾 at varying times



1. During the evening, number of snacks : 🔾 no snacks

🔾 1 snack

🔾 more than 1 snack

1. During the evening, snack times : 🔾 always at the same time

🔾 at the same time for 4 out of 5 days

🔾 at varying times

| 10 | How many days do you eat **snacks** at the weekend (e.g. fruit, biscuits, chocolate)? Please tick the right answer: |
| --- | --- |

1. During the morning on 🔾 0 🔾 1 🔾 2 day(s)
2. During the afternoon on 🔾 0 🔾 1 🔾 2 day(s)
3. During the evening on 🔾 0 🔾 1 🔾 2 day(s)

| 11 | How many **snacks** do you have at the weekend?  Do you eat these snacks at regular or variable times? |
| --- | --- |

1. During the morning, number of snacks : 🔾 no snack

🔾 1 snack

🔾 more than 1 snack

1. Snack times in the morning: 🔾 always at the same time

🔾 at varying times



1. During the afternoon, number of snacks : 🔾 no snacks

🔾 1 snack

🔾 more than 1 snack

1. Afternoon snack times : 🔾 always at the same time

🔾 at varying times



1. During the evening, number of snacks : 🔾 no snacks

🔾 1 snack

🔾 more than 1 snack

1. During the evening, snack times : 🔾 always at the same time

🔾 at varying times

**Part B. Table of eating patterns over the week (Monday - Friday)**

**Hours of sleep and food intake in the morning and afternoon during the week**

|  | **06.00 -06.30** | **06.30 -07.00** | **07.00 -07.30** | **07.30 -08.00** | **08.00 -08.30** | **08.30 -09.00** | **09.00 -09.30** | **09.30 -10.00** | **10.00 -10.30** | **10.30 -11.00** | **11.00 -11.30** | **11.30 -12.00** | **12.00 -12.30** | **12.30 -13.00** | **13.00 -13.30** | **13.30 -14.00** | **14.00 -14.30** | **14.30 -15.00** | **15.00 -15.30** | **15.30 -16.00** | **16.00 -16.30** | **16.30 -17.00** | **17.00 -17.30** | **17.30 -18.00** |
| --- | --- | --- | --- | --- | --- | --- | --- | --- | --- | --- | --- | --- | --- | --- | --- | --- | --- | --- | --- | --- | --- | --- | --- | --- |
|  |  |  |  |  |  |  |  |  |  |  |  |  |  |  |  |  |  |  |  |  |  |  |  |  |
|  |  |  |  |  |  |  |  |  |  |  |  |  |  |  |  |  |  |  |  |  |  |  |  |  |
|  |  |  |  |  |  |  |  |  |  |  |  |  |  |  |  |  |  |  |  |  |  |  |  |  |
| **Wake-up time** | 🔾 | 🔾 | 🔾 | 🔾 | 🔾 | 🔾 | 🔾 | 🔾 | 🔾 | 🔾 | 🔾 | 🔾 | 🔾 | 🔾 | 🔾 | 🔾 | 🔾 | 🔾 | 🔾 | 🔾 | 🔾 | 🔾 | 🔾 | 🔾 |
| **Breakfast** | | | | | | | | | | | | | | | | | | | | | | | | |
| Drinks | 🔾 | 🔾 | 🔾 | 🔾 | 🔾 | 🔾 | 🔾 | 🔾 | 🔾 | 🔾 | 🔾 | 🔾 | 🔾 | 🔾 | 🔾 | 🔾 | 🔾 | 🔾 | 🔾 | 🔾 | 🔾 | 🔾 | 🔾 | 🔾 |
| Food | 🔾 | 🔾 | 🔾 | 🔾 | 🔾 | 🔾 | 🔾 | 🔾 | 🔾 | 🔾 | 🔾 | 🔾 | 🔾 | 🔾 | 🔾 | 🔾 | 🔾 | 🔾 | 🔾 | 🔾 | 🔾 | 🔾 | 🔾 | 🔾 |
| **Lunch** | | | | | | | | | | | | | | | | | | | | | | | | |
| Drinks | 🔾 | 🔾 | 🔾 | 🔾 | 🔾 | 🔾 | 🔾 | 🔾 | 🔾 | 🔾 | 🔾 | 🔾 | 🔾 | 🔾 | 🔾 | 🔾 | 🔾 | 🔾 | 🔾 | 🔾 | 🔾 | 🔾 | 🔾 | 🔾 |
| Food | 🔾 | 🔾 | 🔾 | 🔾 | 🔾 | 🔾 | 🔾 | 🔾 | 🔾 | 🔾 | 🔾 | 🔾 | 🔾 | 🔾 | 🔾 | 🔾 | 🔾 | 🔾 | 🔾 | 🔾 | 🔾 | 🔾 | 🔾 | 🔾 |
| **Evening meal** | | | | | | | | | | | | | | | | | | | | | | | | |
| Drinks | 🔾 | 🔾 | 🔾 | 🔾 | 🔾 | 🔾 | 🔾 | 🔾 | 🔾 | 🔾 | 🔾 | 🔾 | 🔾 | 🔾 | 🔾 | 🔾 | 🔾 | 🔾 | 🔾 | 🔾 | 🔾 | 🔾 | 🔾 | 🔾 |
| Food | 🔾 | 🔾 | 🔾 | 🔾 | 🔾 | 🔾 | 🔾 | 🔾 | 🔾 | 🔾 | 🔾 | 🔾 | 🔾 | 🔾 | 🔾 | 🔾 | 🔾 | 🔾 | 🔾 | 🔾 | 🔾 | 🔾 | 🔾 | 🔾 |
| **Morning and afternoon snacks** | | | | | | | | | | | |  |  |  |  |  |  |  |  |  |  |  |  |  |
| Water | 🔾 | 🔾 | 🔾 | 🔾 | 🔾 | 🔾 | 🔾 | 🔾 | 🔾 | 🔾 | 🔾 | 🔾 | 🔾 | 🔾 | 🔾 | 🔾 | 🔾 | 🔾 | 🔾 | 🔾 | 🔾 | 🔾 | 🔾 | 🔾 |
| Drinks | 🔾 | 🔾 | 🔾 | 🔾 | 🔾 | 🔾 | 🔾 | 🔾 | 🔾 | 🔾 | 🔾 | 🔾 | 🔾 | 🔾 | 🔾 | 🔾 | 🔾 | 🔾 | 🔾 | 🔾 | 🔾 | 🔾 | 🔾 | 🔾 |
| Food | 🔾 | 🔾 | 🔾 | 🔾 | 🔾 | 🔾 | 🔾 | 🔾 | 🔾 | 🔾 | 🔾 | 🔾 | 🔾 | 🔾 | 🔾 | 🔾 | 🔾 | 🔾 | 🔾 | 🔾 | 🔾 | 🔾 | 🔾 | 🔾 |
|  | | | | | | | | | | | | | | | | | | | | | | | | |
| **Daytime naps** | 🔾 | 🔾 | 🔾 | 🔾 | 🔾 | 🔾 | 🔾 | 🔾 | 🔾 | 🔾 | 🔾 | 🔾 | 🔾 | 🔾 | 🔾 | 🔾 | 🔾 | 🔾 | 🔾 | 🔾 | 🔾 | 🔾 | 🔾 | 🔾 |
|  | **06.00 -06.30** | **06.30 -07.00** | **07.00 -07.30** | **07.30 -08.00** | **08.00 -08.30** | **08.30 -09.00** | **09.00 -09.30** | **09.30 -10.00** | **10.00 -10.30** | **10.30 -11.00** | **11.00 -11.30** | **11.30 -12.00** | **12.00 -12.30** | **12.30 -13.00** | **13.00 -13.30** | **13.30 -14.00** | **14.00 -14.30** | **14.30 -15.00** | **15.00 -15.30** | **15.30 -16.00** | **16.00 -16.30** | **16.30 -17.00** | **17.00 -17.30** | **17.30 -18.00** |

**Hours of sleep and food intake in the evening and at night during the week (Monday - Friday)**

|  | **18.00-18.30** | **18.30-19.00** | **19.00-19.30** | **19.30-20.00** | **20.00-20.30** | **20.30-21.00** | **21.00-21.30** | **21.30-22.00** | **22.00-22.30** | **22.30-23.00** | **23.00-23.30** | **23.00-00.00** | **00.00-00.30** | **00.30-01.00** | **01.00-01.30** | **01.30-02.00** | **02.00-02.30** | **02.30-03.00** | **03.00-03.30** | **03.30-04.00** | **04.00-04.30** | **04.30-05.00** | **05.00-05.30** | **05.30-06.00** |
| --- | --- | --- | --- | --- | --- | --- | --- | --- | --- | --- | --- | --- | --- | --- | --- | --- | --- | --- | --- | --- | --- | --- | --- | --- |
|  |  |  |  |  |  |  |  |  |  |  |  |  |  |  |  |  |  |  |  |  |  |  |  |  |
|  |  |  |  |  |  |  |  |  |  |  |  |  |  |  |  |  |  |  |  |  |  |  |  |  |
|  |  |  |  |  |  |  |  |  |  |  |  |  |  |  |  |  |  |  |  |  |  |  |  |  |
| **Evening meal** | | | | | | | | | | | | | | | | | | | | | | | | |
| Drinks | 🔾 | 🔾 | 🔾 | 🔾 | 🔾 | 🔾 | 🔾 | 🔾 | 🔾 | 🔾 | 🔾 | 🔾 | 🔾 | 🔾 | 🔾 | 🔾 | 🔾 | 🔾 | 🔾 | 🔾 | 🔾 | 🔾 | 🔾 | 🔾 |
| Food | 🔾 | 🔾 | 🔾 | 🔾 | 🔾 | 🔾 | 🔾 | 🔾 | 🔾 | 🔾 | 🔾 | 🔾 | 🔾 | 🔾 | 🔾 | 🔾 | 🔾 | 🔾 | 🔾 | 🔾 | 🔾 | 🔾 | 🔾 | 🔾 |
| **Evening and night-time snacks** | | | | | | | | | | | | | | | | | | | | | | | | |
| Water | 🔾 | 🔾 | 🔾 | 🔾 | 🔾 | 🔾 | 🔾 | 🔾 | 🔾 | 🔾 | 🔾 | 🔾 | 🔾 | 🔾 | 🔾 | 🔾 | 🔾 | 🔾 | 🔾 | 🔾 | 🔾 | 🔾 | 🔾 | 🔾 |
| Drinks | 🔾 | 🔾 | 🔾 | 🔾 | 🔾 | 🔾 | 🔾 | 🔾 | 🔾 | 🔾 | 🔾 | 🔾 | 🔾 | 🔾 | 🔾 | 🔾 | 🔾 | 🔾 | 🔾 | 🔾 | 🔾 | 🔾 | 🔾 | 🔾 |
| Food | 🔾 | 🔾 | 🔾 | 🔾 | 🔾 | 🔾 | 🔾 | 🔾 | 🔾 | 🔾 | 🔾 | 🔾 | 🔾 | 🔾 | 🔾 | 🔾 | 🔾 | 🔾 | 🔾 | 🔾 | 🔾 | 🔾 | 🔾 | 🔾 |
|  | | | | | | | | | | | | | | | | | | | | | | | | |
| **Bedtime** | 🔾 | 🔾 | 🔾 | 🔾 | 🔾 | 🔾 | 🔾 | 🔾 | 🔾 | 🔾 | 🔾 | 🔾 | 🔾 | 🔾 | 🔾 | 🔾 | 🔾 | 🔾 | 🔾 | 🔾 | 🔾 | 🔾 | 🔾 | 🔾 |
|  | **18.00-18.30** | **18.30-19.00** | **19.00-19.30** | **19.30-20.00** | **20.00-20.30** | **20.30-21.00** | **21.00-21.30** | **21.30-22.00** | **22.00-22.30** | **22.30-23.00** | **23.00-23.30** | **23.00-00.00** | **00.00-00.30** | **00.30-01.00** | **01.00-01.30** | **01.30-02.00** | **02.00-02.30** | **02.30-03.00** | **03.00-03.30** | **03.30-04.00** | **04.00-04.30** | **04.30-05.00** | **05.00-05.30** | **05.30-06.00** |

**Food rhythm table over the weekend (Saturday and Sunday)**

**Hours of sleep and food intake in the morning and afternoon at the weekend**

|  | **06.00 -06.30** | **06.30 -07.00** | **07.00 -07.30** | **07.30 -08.00** | **08.00 -08.30** | **08.30 -09.00** | **09.00 -09.30** | **09.30 -10.00** | **10.00 -10.30** | **10.30 -11.00** | **11.00 -11.30** | **11.30 -12.00** | **12.00 -12.30** | **12.30 -13.00** | **13.00 -13.30** | **13.30 -14.00** | **14.00 -14.30** | **14.30 -15.00** | **15.00 -15.30** | **15.30 -16.00** | **16.00 -16.30** | **16.30 -17.00** | **17.00 -17.30** | **17.30 -18.00** |
| --- | --- | --- | --- | --- | --- | --- | --- | --- | --- | --- | --- | --- | --- | --- | --- | --- | --- | --- | --- | --- | --- | --- | --- | --- |
|  |  |  |  |  |  |  |  |  |  |  |  |  |  |  |  |  |  |  |  |  |  |  |  |  |
|  |  |  |  |  |  |  |  |  |  |  |  |  |  |  |  |  |  |  |  |  |  |  |  |  |
|  |  |  |  |  |  |  |  |  |  |  |  |  |  |  |  |  |  |  |  |  |  |  |  |  |
| **Wake-up time** | 🔾 | 🔾 | 🔾 | 🔾 | 🔾 | 🔾 | 🔾 | 🔾 | 🔾 | 🔾 | 🔾 | 🔾 | 🔾 | 🔾 | 🔾 | 🔾 | 🔾 | 🔾 | 🔾 | 🔾 | 🔾 | 🔾 | 🔾 | 🔾 |
| **Breakfast** | | | | | | | | | | | | | | | | | | | | | | | | |
| Drinks | 🔾 | 🔾 | 🔾 | 🔾 | 🔾 | 🔾 | 🔾 | 🔾 | 🔾 | 🔾 | 🔾 | 🔾 | 🔾 | 🔾 | 🔾 | 🔾 | 🔾 | 🔾 | 🔾 | 🔾 | 🔾 | 🔾 | 🔾 | 🔾 |
| Food | 🔾 | 🔾 | 🔾 | 🔾 | 🔾 | 🔾 | 🔾 | 🔾 | 🔾 | 🔾 | 🔾 | 🔾 | 🔾 | 🔾 | 🔾 | 🔾 | 🔾 | 🔾 | 🔾 | 🔾 | 🔾 | 🔾 | 🔾 | 🔾 |
| **Lunch** | | | | | | | | | | | | | | | | | | | | | | | | |
| Drinks | 🔾 | 🔾 | 🔾 | 🔾 | 🔾 | 🔾 | 🔾 | 🔾 | 🔾 | 🔾 | 🔾 | 🔾 | 🔾 | 🔾 | 🔾 | 🔾 | 🔾 | 🔾 | 🔾 | 🔾 | 🔾 | 🔾 | 🔾 | 🔾 |
| Food | 🔾 | 🔾 | 🔾 | 🔾 | 🔾 | 🔾 | 🔾 | 🔾 | 🔾 | 🔾 | 🔾 | 🔾 | 🔾 | 🔾 | 🔾 | 🔾 | 🔾 | 🔾 | 🔾 | 🔾 | 🔾 | 🔾 | 🔾 | 🔾 |
| **Evening meal** | | | | | | | | | | | | | | | | | | | | | | | | |
| Drinks | 🔾 | 🔾 | 🔾 | 🔾 | 🔾 | 🔾 | 🔾 | 🔾 | 🔾 | 🔾 | 🔾 | 🔾 | 🔾 | 🔾 | 🔾 | 🔾 | 🔾 | 🔾 | 🔾 | 🔾 | 🔾 | 🔾 | 🔾 | 🔾 |
| Food | 🔾 | 🔾 | 🔾 | 🔾 | 🔾 | 🔾 | 🔾 | 🔾 | 🔾 | 🔾 | 🔾 | 🔾 | 🔾 | 🔾 | 🔾 | 🔾 | 🔾 | 🔾 | 🔾 | 🔾 | 🔾 | 🔾 | 🔾 | 🔾 |
| **Morning and afternoon snacks** | | | | | | | | | | | |  |  |  |  |  |  |  |  |  |  |  |  |  |
| Water | 🔾 | 🔾 | 🔾 | 🔾 | 🔾 | 🔾 | 🔾 | 🔾 | 🔾 | 🔾 | 🔾 | 🔾 | 🔾 | 🔾 | 🔾 | 🔾 | 🔾 | 🔾 | 🔾 | 🔾 | 🔾 | 🔾 | 🔾 | 🔾 |
| Drinks | 🔾 | 🔾 | 🔾 | 🔾 | 🔾 | 🔾 | 🔾 | 🔾 | 🔾 | 🔾 | 🔾 | 🔾 | 🔾 | 🔾 | 🔾 | 🔾 | 🔾 | 🔾 | 🔾 | 🔾 | 🔾 | 🔾 | 🔾 | 🔾 |
| Food | 🔾 | 🔾 | 🔾 | 🔾 | 🔾 | 🔾 | 🔾 | 🔾 | 🔾 | 🔾 | 🔾 | 🔾 | 🔾 | 🔾 | 🔾 | 🔾 | 🔾 | 🔾 | 🔾 | 🔾 | 🔾 | 🔾 | 🔾 | 🔾 |
|  | | | | | | | | | | | | | | | | | | | | | | | | |
| **Daytime naps** | 🔾 | 🔾 | 🔾 | 🔾 | 🔾 | 🔾 | 🔾 | 🔾 | 🔾 | 🔾 | 🔾 | 🔾 | 🔾 | 🔾 | 🔾 | 🔾 | 🔾 | 🔾 | 🔾 | 🔾 | 🔾 | 🔾 | 🔾 | 🔾 |
|  | **06.00 -06.30** | **06.30 -07.00** | **07.00 -07.30** | **07.30 -08.00** | **08.00 -08.30** | **08.30 -09.00** | **09.00 -09.30** | **09.30 -10.00** | **10.00 -10.30** | **10.30 -11.00** | **11.00 -11.30** | **11.30 -12.00** | **12.00 -12.30** | **12.30 -13.00** | **13.00 -13.30** | **13.30 -14.00** | **14.00 -14.30** | **14.30 -15.00** | **15.00 -15.30** | **15.30 -16.00** | **16.00 -16.30** | **16.30 -17.00** | **17.00 -17.30** | **17.30 -18.00** |

**Hours of sleep and food intake in the evening and at night at the weekend (Saturday and Sunday)**

|  | **18.00-18.30** | **18.30-19.00** | **19.00-19.30** | **19.30-20.00** | **20.00-20.30** | **20.30-21.00** | **21.00-21.30** | **21.30-22.00** | **22.00-22.30** | **22.30-23.00** | **23.00-23.30** | **23.00-00.00** | **00.00-00.30** | **00.30-01.00** | **01.00-01.30** | **01.30-02.00** | **02.00-02.30** | **02.30-03.00** | **03.00-03.30** | **03.30-04.00** | **04.00-04.30** | **04.30-05.00** | **05.00-05.30** | **05.30-06.00** |
| --- | --- | --- | --- | --- | --- | --- | --- | --- | --- | --- | --- | --- | --- | --- | --- | --- | --- | --- | --- | --- | --- | --- | --- | --- |
|  |  |  |  |  |  |  |  |  |  |  |  |  |  |  |  |  |  |  |  |  |  |  |  |  |
|  |  |  |  |  |  |  |  |  |  |  |  |  |  |  |  |  |  |  |  |  |  |  |  |  |
|  |  |  |  |  |  |  |  |  |  |  |  |  |  |  |  |  |  |  |  |  |  |  |  |  |
| **Evening meal** | | | | | | | | | | | | | | | | | | | | | | | | |
| Drinks | 🔾 | 🔾 | 🔾 | 🔾 | 🔾 | 🔾 | 🔾 | 🔾 | 🔾 | 🔾 | 🔾 | 🔾 | 🔾 | 🔾 | 🔾 | 🔾 | 🔾 | 🔾 | 🔾 | 🔾 | 🔾 | 🔾 | 🔾 | 🔾 |
| Food | 🔾 | 🔾 | 🔾 | 🔾 | 🔾 | 🔾 | 🔾 | 🔾 | 🔾 | 🔾 | 🔾 | 🔾 | 🔾 | 🔾 | 🔾 | 🔾 | 🔾 | 🔾 | 🔾 | 🔾 | 🔾 | 🔾 | 🔾 | 🔾 |
| **Evening and nighttime snacks** | | | | | | | | | | | | | | | | | | | | | | | | |
| Water | 🔾 | 🔾 | 🔾 | 🔾 | 🔾 | 🔾 | 🔾 | 🔾 | 🔾 | 🔾 | 🔾 | 🔾 | 🔾 | 🔾 | 🔾 | 🔾 | 🔾 | 🔾 | 🔾 | 🔾 | 🔾 | 🔾 | 🔾 | 🔾 |
| Drinks | 🔾 | 🔾 | 🔾 | 🔾 | 🔾 | 🔾 | 🔾 | 🔾 | 🔾 | 🔾 | 🔾 | 🔾 | 🔾 | 🔾 | 🔾 | 🔾 | 🔾 | 🔾 | 🔾 | 🔾 | 🔾 | 🔾 | 🔾 | 🔾 |
| Food | 🔾 | 🔾 | 🔾 | 🔾 | 🔾 | 🔾 | 🔾 | 🔾 | 🔾 | 🔾 | 🔾 | 🔾 | 🔾 | 🔾 | 🔾 | 🔾 | 🔾 | 🔾 | 🔾 | 🔾 | 🔾 | 🔾 | 🔾 | 🔾 |
|  | | | | | | | | | | | | | | | | | | | | | | | | |
| **Bedtime** | 🔾 | 🔾 | 🔾 | 🔾 | 🔾 | 🔾 | 🔾 | 🔾 | 🔾 | 🔾 | 🔾 | 🔾 | 🔾 | 🔾 | 🔾 | 🔾 | 🔾 | 🔾 | 🔾 | 🔾 | 🔾 | 🔾 | 🔾 | 🔾 |
|  | **18.00-18.30** | **18.30-19.00** | **19.00-19.30** | **19.30-20.00** | **20.00-20.30** | **20.30-21.00** | **21.00-21.30** | **21.30-22.00** | **22.00-22.30** | **22.30-23.00** | **23.00-23.30** | **23.00-00.00** | **00.00-00.30** | **00.30-01.00** | **01.00-01.30** | **01.30-02.00** | **02.00-02.30** | **02.30-03.00** | **03.00-03.30** | **03.30-04.00** | **04.00-04.30** | **04.30-05.00** | **05.00-05.30** | **05.30-06.00** |
